# Supplementary material for: Diabetes increases the risk of COVID-19 in an altitude dependent manner: An analysis of 1,280,806 Mexican patients
Source: PLoS One. 2021 Aug 3;16(8):e0255144. doi: 10.1371/journal.pone.0255144 (PMC8330906; doi:10.1371/journal.pone.0255144)
Supplement: Attachment — Submitted filename: Response to reviewers.docx [file pone.0255144.s001.docx]

1. Please ensure that your manuscript meets PLOS ONE's style requirements, including those for file naming. The PLOS ONE style templates can be found at <https://journals.plos.org/plosone/s/file?id=wjVg/PLOSOne_formatting_sample_main_body.pdf> and <https://journals.plos.org/plosone/s/file?id=ba62/PLOSOne_formatting_sample_title_authors_affiliations.pdf>
   a) The style has been corrected
2. In your Methods section, please provide additional information about the data collection methods and the demographic details of your participants. Please ensure you have provided sufficient details to replicate the analyses such as:
   a) the dates the medical records were originally collected,
      i. Data was accesed at Sept 9^th^ 2020. As the Mexican government releases their databases to the public domain with a 24 hour delay, the available registers dated until Sept 8^th^ 2020.
   b) the dates on which you accessed the databases
      i. Now specified in methods: Data was accesed at Sept 9^th^ 2020.
   c) a table of relevant demographic details
      i. Now available as “Table 1”
   d) the institutions that participants were treated at originally.
      i. As the list of healthcare institutions is extensive due to the nature of the database (nation-wide), we specifify in “Methods” that we included all patients treated along the 475 USMERs (Monitoring Units of Viral Respiratory Infections, acronym in spanish)
3. Please amend your list of authors on the manuscript to ensure that each author is linked to an affiliation. Authors’ affiliations should reflect the institution where the work was done (if authors moved subsequently, you can also list the new affiliation stating “current affiliation:….” as necessary).
   a) The style has been corrected
4. Please include a copy of Tables 1 and 2 which you refer to in your text on page 4 and 5.
   a) Tables 1 and 2 are now attached to the manuscript
5. Reviewer #1: p3 ln 92 - constraints of this study = better suited for the discussion section
   a) Constraints were added to the discussion section.
6. p3 ln 100 - ethics etc - name of the committee that Ok-ed the study and date of approval
   a) We wrote: “approval from an institutional ethics committee”
   b) Now it says: “The study received approval from Universidad Peruana Cayetano Heredia’s ethics committee with registration number SIDISI 202908. “
7. Discussion - Less on DM and Covid-19 (which is widely known) - focus on attitude and Covid-19 (which is less well known)
   a) The discussion now focuses on the possible pathophysiology related to our findings.
